# Limited Diagnostic Value of Blood Cultures in Patients with Soft Tissue Infections Transferred to a Quaternary Care Center

**DOI:** 10.3390/bioengineering12060609

**Published:** 2025-06-03

**Authors:** Mira H. Ghneim, Gregory M. Schrank, William Teeter, Brooke Andersen, Anna Brown, Quincy K. Tran

**Affiliations:** 1 Department of Surgery, University of Maryland School of Medicine, Baltimore, MD 21201, USA; 2 Program in Trauma, The R Adam Cowley Shock Trauma Center, University of Maryland School of Medicine, Baltimore, MD 21201, USA; william.teeter@som.umaryland.edu (W.T.); kandersen2@umm.edu (B.A.); anna.brown@umm.edu (A.B.); qtran@som.umaryland.edu (Q.K.T.); 3 Department of Medicine, University of Maryland School of Medicine, Baltimore, MD 21201, USA; gschrank@som.umaryland.edu; 4 Department of Emergency Medicine, University of Maryland School of Medicine, Baltimore, MD 21201, USA; 5 National Study Center for Trauma and EMS, University of Maryland School of Medicine, Baltimore, MD 21201, USA; 6 Research Associate Program in Emergency Medicine & Critical Care, Department of Emergency Medicine, University of Maryland School of Medicine, Baltimore, MD 21201, USA

**Keywords:** soft tissue infections, blood culture, utility, wound culture, transfer

## Abstract

**Introduction:** Patients with soft tissue infection are often encountered in clinical practice. The mainstay of treatment typically includes antimicrobial therapy, followed by surgical debridement when indicated. Blood cultures are often performed prior to starting the first dose of antibiotics. However, when patients require transfer to tertiary/quaternary-level care for more advanced surgical interventions, blood cultures are often repeated despite patients being on broad-spectrum antibiotics. Our study aims to investigate the utility of blood cultures following transfer to a higher level of care. **Methods:** This is a retrospective study involving adult patients (≥18 years of age) who were transferred to a quaternary academic center with soft tissue infections between 15 June 2018 and 15 February 2022. Patients with incomplete medical records and/or without blood culture data after arrival were excluded. The primary outcome was the rate of positive blood cultures post-transfer. Descriptive analyses were performed, and comparisons between groups were expressed as absolute differences and 95% CI. **Results:** We analyzed 303 patients with a mean (+/−SD) age of 54 (14) years, and 199 (66%) were male. Necrotizing soft tissue infections (NSTIs) predominated, 198 patients (65%), with a majority of the NSTIs involving the perineum (112, 37%). The prevalence of positive blood cultures was 20 (7%) for pre-transfer and 14 (5%) for post-transfer. Among post-transfer positive blood cultures, 3 (21%) were coagulase-negative *Staphylococcus aureus,* with 2 (14%) cases each for the blood culture categories of polymicrobial, methicillin-sensitive *Staphylococcus aureus*, and *Enterococcus faecalis*, and 2 (14%) with *Candida species*. Among 112 patients with NSTIs of the perineum, 2 (14%) patients had positive blood cultures post-transfer, compared with 110 (38%) patients with negative blood cultures (difference 24%, 95% CI −0.40, −0.12, *p* < 0.001). **Conclusions:** For patients with soft tissue infection, the prevalence of positive blood culture after arrival at our quaternary care center was low at 5%. Pathogenic cases of positive blood cultures after transfer were polymicrobial, methicillin-sensitive *Staphylococcus aureus* and *Candida* species. However, the low number of post-transfer positive blood cultures limits the strength of the inference and should be interpreted cautiously. Further studies are necessary to confirm our observation. Clinicians at tertiary/quaternary care centers should consider the utility of obtaining blood cultures from patients with soft tissue infections transferred from other facilities.

## 1. Introduction

Non-necrotizing skin and soft tissue infections (nNSTIs), including superficial cellulitis, furuncles, carbuncles, and uncomplicated abscesses, are commonly encountered in clinical practice [1]. These infections are typically caused by regional skin flora or organisms from adjacent mucous membranes and may be polymicrobial [1,2]. However, monomicrobial infections with *Staphylococcus aureus* and Group A *Streptococcus* (GAS) are frequently implicated [1,2]. Diagnosis is often made based on physical examination and laboratory findings, with management focused on appropriate antimicrobial therapy and, when necessary, surgical drainage [1,2].

In contrast, necrotizing soft tissue infections (NSTIs) are life-threatening infections characterized by the rapid destruction of the skin, subcutaneous fat, fascia, and/or muscle [3]. The majority of NSTIs affect the extremities (57–73%), followed by the perineum (13–40%), trunk (13–26%), and head and neck (2–10%) [3]. The most common organisms associated with upper and lower extremity NSTI are GAS or *Staphylococcus aureus*. Less commonly, *Vibrio* spp., *Aeromonas* spp., and *Clostridium* spp. are implicated [3]. Perineal infections (Fournier’s gangrene) are typically polymicrobial, often involving *Escherichia coli*, *Enterococcus* spp., *Bacteroides* spp., and *Pseudomonas* spp. [2,3]. Diagnosis and management of NSTI rely on physical examination, laboratory testing, imaging (computed tomography), and microbiological studies, which typically include blood and deep wound cultures [3]. Management hinges on prompt surgical intervention and empiric broad-spectrum antibiotics [3]. Nearly half of NSTI patients will develop organ failure requiring intensive care unit admission [4], with in-hospital mortality rates ranging from 10–20% [3].

Current guidelines do not recommend routine blood cultures for patients with nNSTI due to their low yield [5,6]. In contrast, blood cultures are recommended for the diagnostic work-up of NSTIs [1,2], though their diagnostic yield remains modest. Available literature suggests that in immunocompetent NSTI patients, blood cultures yield positive results in 10–20% of cases [6], with higher yields (up to 40%) in immunocompromised patients [7].

Blood cultures are frequently obtained at the time of initial presentation and often repeated during the clinical course to assess for persistent bacteremia, identify resistant organisms, or guide antimicrobial therapy. Additionally, the utility of repeat blood cultures, particularly in patients who have already been started on antibiotic treatment and subsequently transferred to a tertiary or quaternary care center, has not been well characterized. Overuse of low-yield diagnostics like repeat blood cultures may contribute to unnecessary healthcare utilization, increased length of stay, and diagnostic uncertainty [8,9,10].

In this study, we aimed to assess the prevalence of positive blood cultures in patients who were transferred to a quaternary care center for the management of necrotizing and non-necrotizing soft tissue infections.

## 2. Methods

### 2.1. Study Design and Patient Selection

After obtaining approval from the institutional review board (IRB# HP-00084554), a retrospective cohort study was conducted in patients (≥18 years of age) presenting with soft tissue infections and admitted to our quaternary-level care center between 15 June 2018 and 15 February 2022. Patients with incomplete medical records and/or without blood culture data after arrival at our quaternary care center were excluded.

### 2.2. Care Pathway for Soft Tissue Infections at Our Quaternary Care Center

Our quaternary care center has a dedicated service, known as the Soft Tissue Service (STS), which specializes in the management of both nNSTIs and NSTIs involving the upper and lower extremities, chest and abdominal wall, decubitus ulcers, and Fournier’s gangrene. The STS team is led by an acute care surgeon and supported by a group of advanced practice providers (APPs), with occasional participation from fellows and surgical residents. The STS accepts both in-state and out-of-state consultations and transfers 24 h a day, 7 days a week.

Once a patient is accepted for transfer by the STS, they are transferred to our critical care resuscitation unit (CCRU). The CCRU is staffed by emergency medicine critical care physicians and supported by a group of critical care APPs. This unit specializes in providing expeditious transfer and resuscitation to critically ill patients with time-sensitive medical and surgical diagnoses.

Upon arrival of a patient with a soft tissue infection to the CCRU, they are evaluated by the CCRU and STS team, and a shared decision is made regarding further resuscitation and indication and timing of operative management. Additional work-up in the CCRU includes baseline labs, blood cultures, and any additional imaging if deemed necessary.

Finally, the patient is also evaluated, preoperatively or post-operatively, by the infectious disease (ID) team, which provides guidance regarding the appropriate antibiotic regimen. The ID team also contacts the referring hospitals to obtain further information regarding blood cultures or wound culture results, which are usually not available to either the CCRU or the STS team members at the time of patients’ arrival at our quaternary care center.

### 2.3. Covariates and Outcomes

The data for this study were collected from the electronic medical records in accordance with the guidelines for retrospective studies [11]. The research team members, who were not blinded to the study hypothesis, were trained by the senior investigator (QT). The research team was trained with sets of 10 patients until the inter-rater agreement reached ≥90% between the team members. During the data collection process, the senior investigator performed random data checks on 10% of the data to ensure accuracy and reliability.

Data collected included patient demographics, past medical history (body mass index [BMI], chronic kidney disease [CKD], history of intravenous drug use, and history of methicillin-resistant *Staphylococcus aureus* infections), and the type of soft tissue infection. BMI was calculated as weight in kilograms divided by height in meters squared (kg/m^2^). BMI categories were classified according to the World Health Organization (WHO) criteria: underweight (<18.5 kg/m^2^), normal weight (18.5–24.9 kg/m^2^), overweight (25.0–29.9 kg/m^2^), and obesity (≥30.0 kg/m^2^) [12]. CKD was defined according to the Kidney Disease: Improving Global Outcomes (KDIGO) clinical practice guidelines as either evidence of kidney damage (e.g., albuminuria, structural abnormalities) or a sustained reduction in estimated glomerular filtration rate (eGFR <60 mL/min/1.73 m^2^) for at least 3 months, irrespective of the underlying cause [13]. History of MRSA was defined as any prior infection or colonization with MRSA noted in the patient’s medical record.

Laboratory values at the time of admission to our center (serum lactate, hemoglobin levels, hemoglobin A1C), blood and wound cultures obtained at our center, organisms identified in blood and wound cultures from our institution, and blood culture results from the transferring institution were collected. Blood culture isolates were classified as either pathogenic or commensal. Organisms were defined as commensals if they appeared on the Centers for Disease Control and Prevention/National Healthcare Safety Network (CDC/NHSN) list of common commensal organisms [14]. For blood cultures that yielded a commensal organism, we assessed for possible contamination by comparing blood culture results with available wound or tissue culture data. In the absence of concordance between culture sources or other clinical indicators of systemic infection, the blood culture isolate was considered likely to represent contamination rather than true bloodstream infection. All wound cultures included in this study were obtained intraoperatively in the operating room during surgical debridement procedures. Specimens were collected from deep tissue using sterile technique to ensure accurate microbiological representation of the infected site.

Data regarding disease severity scores, treatment and hospital course, hospital length of stay (HLOS), discharge disposition, and in-hospital mortality were also collected. Disease severity was assessed using the Sequential Organ Failure Assessment (SOFA) [15] score and Laboratory Risk Indicator for Necrotizing Fasciitis (LRINEC) [16] score. Treatment and hospital course included surgical debridement, extremity amputations, and utilization of hyperbaric treatments.

At our institution, all patients admitted with a diagnosis of NSTI, as determined by the primary surgical service, undergo consultation by the hyperbaric medicine team to evaluate eligibility for adjunctive hyperbaric oxygen therapy (HBOT). Patients are considered eligible for HBOT if they have an active NSTI that has undergone surgical debridement. Contraindications to HBOT include untreated pneumothorax, severe agitation or behavioral disturbance that precludes safe chamber treatment, and hemodynamic instability. Eligibility and contraindications are assessed collaboratively by the hyperbaric and critical care teams on a case-by-case basis. HBOT is typically initiated within 24 h of surgical intervention, with additional sessions provided based on clinical status and surgical findings.

The primary outcome was the rate of positive blood cultures after the patients were transferred to our quaternary care center. The secondary outcome was the rates of positive blood cultures at the referring institution.

### 2.4. Statistical Analysis

We performed descriptive analyses using means (± standard deviations) for continuous variables and percentages for categorical variables. For comparisons of continuous variables, we used Student *t*-tests, with results expressed as differences in means. Chi-square tests were used to compare categorical variables, with results reported as differences in proportions. A post hoc power analysis using https://clincalc.com/stats/power.aspx, accessed on 25 May 2025) was performed to estimate the power (with alpha = 0.05) to detect the differences in blood culture positivity before and after transfer. A *p* value < 0.05 was considered statistically significant. Statistical analyses were performed using Minitab version 4.0 (www.minitab.com, accessed on 5 January 2025; State College, PA, USA).

## 3. Results

### 3.1. Overall Characteristics of the Patient Population

A total of 305 patients were treated for soft tissue infections at our institution between 2018 and 2022. Two patients were excluded from the analysis as blood cultures were not obtained; their families elected to discontinue life-sustaining measures after arrival due to the severity of illness. Among the 303 patients included in the analysis, the mean (SD) age was 54 (14) years, the majority were male (66%), and patients were obese with a BMI of 33 (12) (Table 1).

One hundred and sixty-four (54%) were smokers, 31 (10%) were persons who inject drugs, and 35 (12%) presented with a pre-existing history of MRSA infection/colonization. The mean SOFA score was 5 (5), and the LRINEC score was 7(3) (Table 1).

One hundred and ninety-eight patients presented with NSTIs (65%), with Fournier’s gangrene (37%) being the most frequent subtype, followed by lower extremity NSTIs (20%), chest/abdominal wall NSTs (8%), and upper extremity NSTIs (5%) (Table 1).

Among the 303 patients, 20 (7%) had positive blood cultures obtained at the transferring hospital, none of whom had positive results on repeat testing at our institution. In contrast, 14 (5%) patients had positive blood cultures drawn at our institution, despite initially negative cultures at the transferring facility. Eighty-five (28%) of the patients underwent their index surgical debridement at the referring facility prior to transfer (Table 2).

Following transfer, debridement was performed in 276 (91%) patients, and 110 (36%) patients received HBOT. The mean HLOS was 15 (13) days. At discharge, 139 patients (46%) were discharged home, 46 (15%) were discharged to acute rehabilitation, 92 (30%) to a skilled nursing facility, and 26 patients (9%) died in-hospital or were discharged to hospice (Table 2).

### 3.2. Characteristics, Treatment, and Outcomes for Patients with Post-Transfer Positive Blood Cultures vs. Negative Blood Cultures at Our Quaternary Care Center

Demographics, baseline medical history, and severity of illness were similar between the two groups. Patients with positive blood cultures were more likely to have a history of MRSA colonization or infection (35% vs. 10%; *p* = 0.05). They also had significantly higher white blood cell counts at presentation (19 vs. 14 K/mcL; *p* = 0.02) (Table 1).

The type of soft tissue infection differed between groups. Patients with positive blood cultures were more likely to present with infected decubitus ulcers (21% vs. 5%; *p* = 0.04), whereas those with negative blood cultures were more likely to present with Fournier’s gangrene (38% vs. 14%; *p* < 0.001) (Table 1).

Treatment modalities at our institution were similar between groups. Post-transfer surgical debridement was performed in 86% and 91% of patients in the negative blood culture and positive blood culture groups, respectively. HBOT was administered in 21% of patients with positive blood cultures and 37% of those with negative cultures (*p* = 0.17) (Table 2).

Positive wound cultures were documented in 226 patients (75%) overall. Among those with positive blood cultures, 9 patients (64%) had a positive wound culture, compared with 219 patients (75%) in the negative blood culture group (*p* = 0.36) (Table 2).

The mean HLOS was 14 days (11) for patients with positive blood cultures and 16 days (14) for those with negative cultures (*p* = 0.67) (Table 2). Hospital disposition differed between the two groups. Only 3 patients (21%) in the positive blood culture group were discharged home, compared with 136 patients (47%) in the negative culture group (*p* = 0.02). Discharges to acute rehabilitation (21% vs. 15%; *p* = 0.56), skilled nursing facilities (36% vs. 30%; *p* = 0.67), and in-hospital mortality or hospice discharge (21% vs. 8%; *p* = 0.22) were not significantly different (Table 2).

### 3.3. Microorganisms Identified in Blood and Wound Cultures in Patients Presenting with Positive Blood Cultures from the Transferring and Negative Blood Cultures at Our Quaternary Care Center

Among the 20 patients transferred with positive blood cultures, the most frequently isolated organism was methicillin-sensitive *Staphylococcus aureus* (MSSA), identified in 4 patients (20%), followed by *Streptococcus agalactiae* (Group B Streptococcus) in 2 patients (10%). Other Gram-positive cocci (GPCs) included unspecified coagulase-negative *Staphylococcus*, MRSA, *Staphylococcus capitis*, *Enterococcus faecalis*, alpha-hemolytic streptococci, and beta-hemolytic *Streptococcus* (Group F). Gram-negative rods (GNRs) isolated included *Pseudomonas aeruginosa*, *Klebsiella* species, *Serratia marcescens*, *Vibrio vulnificus*, *Escherichia coli*, *Proteus mirabilis*, *Providencia* species, and an unspecified GNR. Anaerobes and opportunistic pathogens identified included *Prevotella* species, *Fusobacterium nucleatum*, *Trueperella bernardiae*, and *Eikenella corrodens* (Table 3).

Fourteen of the 20 patients had positive wound cultures (70%). Concordant organisms, matching those found in blood cultures, were present in 5 patients (36%), including MSSA or MRSA (n = 3), *Pseudomonas aeruginosa* (n = 1), and *Candida albicans* (n = 1). Polymicrobial wound infections were found in 6 patients (43%), particularly in those with GPC or enteric bloodstream infections. Additional organisms not identified in blood cultures were detected in 13 patients (93%), including anaerobes (*Fusobacterium nucleatum*, *Prevotella* species, *Trueperella bernardiae*), enteric flora (*Escherichia coli*, *Bacteroides fragilis, Morganella morganii*), and *Acinetobacter* species (Table 3).

The most common soft tissue infections among the 20 patients transferred with positive blood cultures were necrotizing soft tissue infections of the lower extremities and abdomen, observed in 10 patients (50%). This was followed by decubitus ulcers in 6 patients (30%) and Fournier’s gangrene in 4 patients (20%) (Table 3).

### 3.4. Microorganisms Identified in Blood and Wound Cultures in Patients Presenting with Positive Blood Cultures from the Transferring and Postive Blood Cultures at Our Quaternary Care Center

Among the 14 patients who presented with positive blood cultures at our institution, the most frequently identified organisms included GPCs—*Coagulase-negative Staphylococcus* (n = 3), *Enterococcus faecalis* (n = 2), methicillin-sensitive *Staphylococcus aureus* (MSSA), methicillin-resistant *Staphylococcus aureus* (MRSA), and *Staphylococcus epidermidis*. Gram-negative and other organisms identified in blood cultures included *Serratia marcescens*, *Sphingomonas* species, *Acinetobacter baumannii*, and Diphtheroids. Fungal isolates included *Candida albicans* and *Candida lusitaniae*. One patient had a polymicrobial bloodstream infection with *Streptococcus agalactiae* (Group B), *Bacteroides fragilis*, and *Staphylococcus* species (Table 4).

Wound cultures were positive in 10 of the 14 patients (71%). Concordant organisms were identified in several cases, including *Enterococcus faecalis*, *Serratia marcescens*, *Candida albicans*, and *Candida lusitaniae*. Additional organisms identified only in wound cultures included *Enterococcus avium*, *Clostridium sporogenes*, *Bacteroides vulgatus*, *Klebsiella pneumoniae*, *Proteus mirabilis*, *Proteus vulgaris*, *Citrobacter freundii*, *Streptococcus anginosus*, *Prevotella* species, and *Bacteroides* species (Table 4).

Fournier’s gangrene was the most common type of soft tissue infection, present in 7 of 14 patients (50%). Lower extremity NSTIs were observed in 3 patients (21%), while upper extremity NSTIs and chest NSTIs were each identified in 2 patients (14%). Debridement at the referring hospital prior to transfer was performed in 6 of the 14 patients (43%) (Table 4).

### 3.5. Wound Culture Results of the Entire Cohort

**Figure 1:**
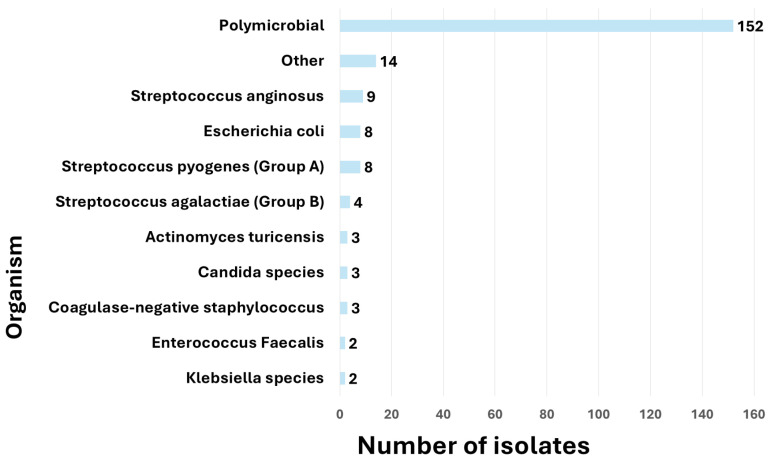
Distribution of wound culture organisms in patients with soft tissue infections, (n = 204). One patient may have more than one organism.

Among the 204 wound cultures isolated from this cohort, the majority were polymicrobial (n = 152, 74%). GPCs accounted for a notable proportion, including *coagulase-negative Staphylococcus* (n = 3, 1%), *Enterococcus faecalis* (n = 2, 1%), and various *Streptococcus* species such as Group A (n = 8, 4%) and Group B (n = 4, 2%). GNRs were less frequent and included *Escherichia coli* (n = 8, 4%), *Klebsiella* species (n = 2, 1%), *Serratia marcescens* (n = 1, <1%), and *Pseudomonas aeruginosa* (n = 1, <1%). Fungal isolates, primarily *Candida* species, were identified in 3 cases (1%). Rare anaerobic organisms such as *Actinomyces turicensis*, *Bacteroides fragilis*, and *Clostridium septicum* were also detected (Figure 1).

## 4. Discussion

In this retrospective study of 303 patients with soft tissue infections admitted to a quaternary referral center for care, we found an overall blood culture positivity rate of 11%, but a lower positivity rate of 5% for blood culture testing performed after arrival at our institution from the referring hospital. Notably, of the 14 positive blood cultures collected after transfer, five were positive for common commensal pathogens, reducing the true positive rate of post-transfer blood cultures to 3%. Interestingly, a higher rate of blood culture positivity post-transfer was observed among patients with infected decubitus ulcers. This may be due to the more indolent, chronic nature of these infections, which could contribute to delays in presentation or potentially differences in the pre-transfer antimicrobial management. The exact cause of this finding remains uncertain, however, and further investigation should be undertaken to determine if it can be replicated.

The overall positivity rate for wound cultures among the cohort was 75%, indicating that surgical debridement with tissue sampling is diagnostically high yield and provides important data to inform treatment decisions for most patients with complicated soft tissue infections. Among the positive blood cultures obtained after transfer, however, potentially four patients (1.3%) had blood culture results with an organism for which identification in blood could potentially alter the diagnostic and treatment plan for the patient: *Staphylococcus aureus* (n = 2) and *Candida* spp. (n = 2). Our findings suggest that for patients with soft tissue infections, obtaining repeat blood cultures after transferring to a referral center are generally low-value tests.

The overall positivity rate of blood cultures in this study aligns with prior research on this topic. A retrospective analysis of a Dutch multicenter study that included 334 patients found a blood culture positivity rate of 16%; however, testing was not performed in almost half of the study population, and as many patients without blood cultures were less severely ill, the overall diagnostic yield would likely be lower if these patients had undergone testing [17]. Blood culture positivity was associated with higher baseline comorbidity. In another single-center observational study of 246 patients with skin and soft tissue infections admitted through an emergency department, only 86 underwent blood culture testing, of whom 7% had a positive result. Neither a reported history of injection drug use nor the presence of fever was associated with a positive blood culture in their study cohort, underscoring the challenge of identifying appropriate clinical indications for blood culture testing in this population [5]. Neither study, however, examined the additional diagnostic value of repeat blood culture testing after transfer, which is a unique clinical question answered by our analysis—and one commonly encountered at large referral centers that care for high-acuity skin and soft tissue infections.

A quite diverse array of pathogens was represented within the positive blood culture results from both the referral hospital and our facility, reflecting the polymicrobial nature of many complex skin and soft tissue infections. Indeed, approximately two-thirds of patients in our study had confirmation of a polymicrobial infection by wound culture results. Also of importance in our findings was the high rate of blood culture contamination seen among the blood cultures obtained after transfer; 36% of positive blood cultures contained a common commensal organism. With an overall contamination rate of 1.7%, which is below the recommended benchmark of 3% [18], however, recent guidance from the Clinical and Laboratory Standards Institute has suggested this benchmark be lowered to a target of <1% [19]. Our study’s findings confirm the need to reinforce best practices to avoid contamination during the process of culture collection but also suggest that the pre-test probability of bacteremia or fungemia in the cohort was low and that over-testing contributed to a high false positivity rate in relation to true positivity.

There is increasing interest in examining the diagnostic value of blood culture testing in different clinical scenarios and patient populations, given the high intensity of testing that occurs in hospitalized patients and the consequences of either false positive or uncertain results, including exposure to antibiotic therapy, increased hospital length of stay, additional diagnostic procedures, and costs incurred from each of these [20,21]. A recent large analysis of medical and surgical inpatient units across 48 hospitals found an overall blood culture positivity rate of approximately 6%, with contamination rates ranging from 1–1.5% [22]. Establishment of benchmarks of utilization and positivity will inform facilities as they consider efforts to refine their practices and increase the yield of blood culture testing for skin and soft tissue patients admitted to their facilities.

## 5. Limitations

Our study has several limitations. First, we lacked information on the timing and type of antibiotic administration at the transferring facilities, which may have influenced blood culture results and limited our ability to assess their diagnostic utility fully. The timing, spectrum, and appropriateness of antimicrobial therapy are critical factors known to influence the diagnostic yield of blood cultures, and the absence of this information limits the interpretability of our findings. Second, because nearly all patients were transferred from a variety of outside hospitals and post-transfer managed by a dedicated soft tissue infection team at a single quaternary care center, our findings may not be generalizable to institutions without similar clinical resources or transfer patterns. Although our team actively coordinated with referring hospitals to gather culture results from their facilities, there may have been incomplete or unavailable data, introducing potential information bias. Third, our post hoc power analysis showed that our study had 18% power to detect the difference between positive blood cultures prior to and post-transfer.

Additionally, the relatively small number of patients with positive blood cultures either before or after transfer limited the statistical power of our comparisons and precluded more advanced analyses such as multivariable regression. In particular, the low number of post-transfer positive events (n = 14) would have rendered any regression model unstable and prone to overfitting, limiting its interpretability and validity. As such, we relied on descriptive and univariate analyses to present our findings, and we acknowledge this as a methodological limitation.

## 6. Conclusion

In this cohort of patients with soft tissue infections transferred to a quaternary care center, the prevalence of post-transfer positive blood cultures was low (5%). The most frequently identified pathogenic organisms included polymicrobial flora, methicillin-sensitive *Staphylococcus aureus*, and *Candida* species. These findings suggest that the diagnostic utility of obtaining additional blood cultures following interfacility transfer may be of limited value. Future studies should focus on optimizing diagnostic strategies to reduce unnecessary testing and better allocate resources toward high-yield assessment tools.

## Figures and Tables

**Table 1:** Demographics, severity of illness, and clinical characteristics of patients presenting with soft tissue infection and post-transfer blood culture assessment at our quaternary care center, (n = 303).

	All Patients (n = 303)	UMMC Blood Cultures (Positive) (n = 14)	UMMC Blood Cultures (Negative) (n = 289)	*p*-Value
Age, years, mean (SD)	54 (14)	50 (16)	54 (14)	0.32
Male, n (%)	199 (66)	6 (43)	193 (67)	0.08
**Past medical history**				
BMI, mean (SD)	33 (12)	27 (9)	33 (12)	0.07
Chronic kidney disease, n (%)	29 (10)	1 (7)	28 (10)	0.72
Smoking, n (%)	164 (54)	7 (50)	157 (54)	0.75
PWID, n (%)	31 (10)	4 (28)	27 (9)	0.11
History of MRSA, n (%)	35 (12)	5 (35)	30 (10)	0.05
**Severity of illness, mean (SD)**				
SOFA	5 (5)	4 (4)	11(4)	0.65
LRINEC	7 (3)	7 (2)	14 (5)	0.40
**Type of soft tissue infection, n (%)**				
Abscess	11 (4)	0 (0)	11 (4)	-
Infected decubitus ulcer	17 (6)	3 (21)	14 (5)	0.04
Myositis	4(1)	0 (0)	4 (1)	-
Osteomyelitis	4 (1)	0 (0)	4 (1)	-
Soft tissue infections, any	69 (23)	5 (36)	64 (33)	0.32
Necrotizing soft tissue infections *	198 (65)	6 (43)	192 (66)	0.09
Chest/abdomen	23 (8)	1(7)	22 (8)	0.99
Upper extremity	16 (5)	1(7)	15 (5)	0.77
Lower extremity	60 (20)	3 (21)	57 (20)	0.99
Perineum	112 (37)	2 (14)	110 (38)	0.001
**Laboratory values, mean (SD)**				
White blood cell count (K/mcl)	19 (11)	14 (6)	19 (11)	0.02
Lactate (mmol/L)	2 (2)	3 (1)	2 (2)	0.35
Hgb-A1C (% total Hgb)	8 (3)	7 (3)	8 (3)	0.45
Hemoglobin(g/dL)	10 (2)	9 (2)	10 (2)	0.04
Sodium mmol/L	135 (4)	135 (6)	135 (4)	0.99
Creatinine (mg/dL)	1.7 (1.8)	1.2 (1)	1.7 (1)	0.15
Glucose (mg/dL)	179 (106)	162 (112)	180 (106)	0.57

* One patient might have infection in more than one anatomical site. UMMC: University of Maryland Medical Center; BMI: body mass index; PWID: patients who inject drugs; MRSA: methicillin resistant *Staphylococcus aureus*; SOFA: sequential organ failure assessment score; LRINEC: Laboratory Risk Indicator for Necrotizing Fasciitis; Hgb: hemoglobin; HLOS: hospital length of stay. Normal ranges for White blood cell count (4.5–11), lactate (0.5–2.2), Hgb-A1C (normal < 5.7%, prediabetes: 5.7% to 6.4%, diabetes ≥ 6.5%), Hgb (12.6–17.4), sodium (136–145), creatinine (0.66–1.25), glucose (70–99).

**Table 2:** Management and outcomes of patients presenting with soft tissue infection and post-transfer blood culture assessment at our quaternary care center, (n = 303).

	All Patients (n = 303)	UMMC Blood Cultures (Positive) (n = 14)	UMMC Blood Cultures (Negative) (n = 289)	*p*-Value
**Transferring facility data, n (%)**				
Positive blood cultures	20 (7)	0 (0)	20 (7)	0.001
Debridement prior to transfer	85 (28)	4 (29)	81 (28)	0.97
Positive wound cultures, n (%)	226 (75)	9 (64)	219 (75)	0.36
**Management at the admitting facility**				
Debridement	276 (91)	12 (86)	264 (91)	0.36
Hyperbaric oxygen therapy, n (%)	110 (36)	3 (21)	107 (37)	0.17
Major amputation, n (%)	42 (14)	3 (21)	39 (13)	0.48
Hospital length of stay, mean (SD)	15 (13)	14 (11)	16 (14)	0.67
**Hospital disposition, n (%)**				
Home	139(46)	3 (21)	136 (47)	0.02
Acute rehab	46 (15)	3 (21)	43 (15)	0.56
Skilled nursing facility	92 (30)	5 (36)	87 (30)	0.67
Dead/hospice	26 (9)	3 (21)	23 (8)	0.22

**Table 3:** Microorganisms identified in blood and wound cultures from patients transferred with positive blood cultures *(n = 20)*.

Organisms from Blood Cultures	Organisms from Wound Cultures	Soft Tissue Infection Type
Coagulase-negative *Staphylococcus*	*Diphtheroids* *	Decubitus ulcer
Methicillin-sensitive *Staphylococcus aureus*	None	Lower extremity NSTI
Methicillin-sensitive *Staphylococcus aureus*	Methicillin-sensitive *Staphylococcus aureus*	Fournier’s gangrene
Methicillin-sensitive *Staphylococcus aureus*	Methicillin-sensitive *Staphylococcus aureus*	Lower extremity NSTI
Methicillin-sensitive *Staphylococcus aureus*	Polymicrobial	Lower extremity NSTI
Methicillin-resistant *Staphylococcus aureus*	Methicillin-resistant *Staphylococcus aureus*	Fournier’s gangrene
*Staphylococcus capitis*	*Streptococcus dysgalactiae* *Fusobacterium nucleatum* *Trueperella bernardiae*	Lower extremity NSTI
Alpha-hemolytic *Streptococcus*	Polymicrobial	Fournier’s gangrene
Beta-hemolytic *Streptococcus, Group F*	None	Lower extremity NSTI
*Streptococcus agalactiae*	None	Decubitus ulcer
*Streptococcus constellatus*	*Escherichia coli* *Streptococcus constellatus* *Bacteroides fragilis* Enteric flora	Lower extremity NSTI
*Micrococcus luteus*	*Eikenella corrodens* *Streptococcus anginosus* *Enterococcus avium*	Abdomen NSTI
*Serratia marcescens*	None	Lower extremity NSTI
*Vibrio vulnificus*	None	Lower extremity NSTI
*Pseudomonas aeruginosa*	*Pseudomonas aeruginosa, Morganella morganii*	Fournier’s gangrene
*Klebsiella* species	*Escherichia coli, Pseudomonas aeruginosa*	Fournier’s gangrene
*Prevotella* species	*Escherichia coli* *Providencia species* *Enterococcus faecalis* *Enterococcus faecium* *Enterococcus raffinosus* multidrug-resistant *Acinetobacter* spp.	Fournier’s gangrene
Unspecified Gram-negative rod	None	Decubitus ulcer
*Escherichia coli* *Proteus mirabilis* Coagulase-*negative Staphylococcus*	*Escherichia coli, Proteus mirabilis*	Decubitus ulcer
*Candida albicans*	*Candida albicans, Staphylococcus aureus*	Fournier’s gangrene

* Diphtheroids, which refers to Gram-positive bacilli, *Corynebacterium* spp., is currently reported by our Institution’s microbiology specialists.

**Table 4:** Organisms identified in blood cultures obtained at our institution and corresponding wound cultures in patients with soft tissue infections and negative blood cultures at the transferring facility (n = 14).

Organisms from Blood Cultures	Organisms from Wound Cultures	Soft Tissue Infection Type	Debridement at the Referring Center
MSSA	None	Lower extremity NSTI	Yes
Coagulase-negative *Staphylococcus*	*Enterococcus avium* *Clostridium sporogenes* *Bacteroides vulgatus*	Fournier’s gangrene	No
Coagulase-negative *Staphylococcus*	None	Lower extremity NSTI	No
Coagulase-negative *Staphylococcus*	None	Fournier’s gangrene	Yes
MRSA	None	Chest NSI and Fournier’s gangrene	No
*Enterococcus faecalis*	*Enterococcus faecalis,* *Pseudomonas aeruginosa*	Lower extremity NSTI	No
*Enterococcus faecalis*	*Enterococcus faecalis,* *Klebsiella pneumoniae*	Fournier’s gangrene	No
*Sphingomonas species*	*Enterococcus faecalis,* *Proteus mirabilis,* *Candida albicans*	Fournier’s gangrene	Yes
*Serratia marcescens*	*Enterococcus faecalis,* *Serratia marcescens*	Fournier’s gangrene	No
*Diphtheroids*	*Acinetobacter baumannii* *Proteus mirabilis* *Staphylococcus epidermidis*	Fournier’s gangrene	Yes
*Candida albicans*	*Citrobacter freundii* *Streptococcus anginosus* *Enterococcus faecalis* *Proteus vulgaris*	Upper extremity NSTI	Yes
Candida lusitaniae *	*Candida lusitaniae* *Candida albicans*	Upper extremity NSTI	No
*Polymicrobial:* *Streptococcus agalactiae (Group B)* *Bacteroides fragilis* *Staphylococcus species*	*Prevotella* *Bacteroides* *Candida albicans*	Fournier’s gangrene	Yes
*Acinetobacter baumannii*	None	Chest NSTI	No

* *Candida lusitaniae*, which refers to called *Clavispora lusitaniae*, currently reported by our Institution’s microbiology specialists.

## Data Availability

No data is available due to privacy restrictions.
